# COVID-19–Related Outcomes in Primary Mitochondrial Diseases: An International Study

**DOI:** 10.1212/WNL.0000000000200240

**Published:** 2022-04-05

**Authors:** Chiara Pizzamiglio, Pedro M. Machado, Rhys H. Thomas, Gráinne S. Gorman, Robert McFarland, Michael G. Hanna, Robert D. S. Pitceathly, on behalf of the MitoCOVID-19 Study Group

**Affiliations:** From the Department of Neuromuscular Diseases (C.P., P.M.M., M.G.H., R.D.S.P.), UCL Queen Square Institute of Neurology and The National Hospital for Neurology and Neurosurgery, London; and Wellcome Centre for Mitochondrial Research (R.H.T., G.S.G., R.M.), Translational and Clinical Research Institute, Faculty of Medical Sciences, Newcastle University, Newcastle upon Tyne, UK.

## Abstract

**Background and Objectives:**

To identify factors associated with severe coronavirus disease 2019 (COVID-19), defined by hospitalization status, in patients with primary mitochondrial diseases (PMDs), thereby enabling future risk stratification and informed management decisions.

**Methods:**

We undertook a cross-sectional, international, registry-based study. Data were extracted from the International Neuromuscular COVID-19 Database and collected between May 1, 2020, and May 31, 2021. The database included subjects with (1) PMD diagnosis (any age), clinically/histopathologically suspected and/or genetically confirmed; and (2) COVID-19 diagnosis classified as “confirmed”, “probable”, or “suspected” based on World Health Organization definitions. The primary outcome was hospitalization because of COVID-19. We collected demographic information, smoking status, coexisting comorbidities, outcomes after COVID-19 infection, and PMD genotype-phenotype. Baseline status was assessed using the modified Rankin scale (mRS) and the Newcastle Mitochondrial Disease Adult Scale (NMDAS).

**Results:**

Seventy-nine subjects with PMDs from 10 countries were included (mean age 41.5 ± 18 years): 25 (32%) were hospitalized, 48 (61%) recovered fully, 28 (35%) improved with sequelae, and 3 (4%) died. Statistically significant differences in hospitalization status were observed in baseline status, including the NMDAS score (*p* = 0.003) and mRS (*p* = 0.001), presence of respiratory dysfunction (*p* < 0.001), neurologic involvement (*p* = 0.003), and more than 4 comorbidities (*p* = 0.002). In multivariable analysis, respiratory dysfunction was independently associated with COVID-19 hospitalization (odds ratio, 7.66; 95% CI, 2–28; *p* = 0.002).

**Discussion:**

Respiratory dysfunction is an independent risk factor for severe COVID-19 in PMDs while high disease burden and coexisting comorbidities contribute toward COVID-19–related hospitalization. These findings will enable risk stratification and informed management decisions for this vulnerable population.

Despite their variable clinical manifestations and severity, people with primary mitochondrial diseases (PMDs) were considered at high risk for serious coronavirus disease 2019 (COVID-19), given the potential for multisystemic involvement and metabolic decompensation during intercurrent illness.^1^ Consequently, people with PMDs were grouped worldwide within the COVID-19 clinically highest risk category (designated “clinically extremely vulnerable” by NHS England, United Kingdom [UK], and “higher risk of severe COVID-19 illness”, by the Centers for Disease Control and Prevention, USA)^2^ and advised to implement the shielding approach, limiting contact with the general population when SARS-CoV-2 infection rates were high.^3^

In this cross-sectional, international, registry-based study, we aimed to identify factors linked with severe COVID-19 in PMDs, thereby enabling future risk stratification and informed management decisions.

## Methods

The NHS England Highly Specialized Services for Rare Mitochondrial Disorders in London and Newcastle, UK, designed a PMD-specific module within the “International Neuromuscular COVID-19 Database”^4^; this was hosted by University College London (see eMethods in the Supplement, links.lww.com/WNL/B828) to record details concerning PMD genotype-phenotype and COVID-19 during the pandemic. Inclusion criteria were (1) a PMD diagnosis (any age) in the opinion of the recruiting healthcare provider, clinically/histopathologically suspected and/or genetically confirmed; and (2) a COVID-19 diagnosis stratified as “confirmed,” “probable,” or “suspected” based on World Health Organization (WHO) definitions.^5^ Baseline status was assessed using the modified Rankin scale (mRS) and the Newcastle Mitochondrial Disease Adult Scale (NMDAS; see eMethods in the Supplement). Key comorbidities included respiratory dysfunction, mitochondrial diabetes (a distinct monogenic form of diabetes mellitus that is specific to PMDs and has been associated with a variety of mitochondrial and nuclear DNA sequence variations),^6,7^ hypertension or other cardiovascular diseases, obesity (body mass index ≥ 30), and neurologic involvement. Respiratory dysfunction was defined by the presence of at least one of the following: (1) obstructive lung disease (chronic obstructive pulmonary disease or asthma), (2) restrictive lung disease, (3) obstructive sleep apnea, (4) use of noninvasive ventilation, and (5) tracheostomy. Neurologic involvement was determined by the presence of at least one of the following: (1) dysphagia, (2) epilepsy, (3) learning disabilities, (4) polyneuropathy, (5) skeletal muscle weakness, and (6) stroke/stroke-like episodes. All comorbidities included under the “neurologic involvement” section were reported as disease-specific neurologic features in the mitochondrial disease module of the database. Only anonymized, nonidentifiable data were collected; thus, participant consent was not required according to the UK Health Research Authority. Statistical analyses were performed using SPSS v19.0 (SPSS Inc). *p* value < 0.05 was considered significant. The primary outcome of this study was hospitalization status for COVID-19. See the eMethods in the Supplement for further details concerning database design and data storage, protocol approvals, and participants.

### Data Availability

Dr. Pizzamiglio had full access to all the data in the study and takes responsibility for the integrity of the data and the accuracy of the data analysis.

## Results

From May 1, 2020, to May 31, 2021, 79 patients (mean age 41.5 years; female 46, 58%, White ethnicity 64, 83%) across 10 countries (UK 44, 56%, Table 1) were registered. Hospitalization status was recorded for all entries; thus, no cases were excluded. Almost one-third of the patients were hospitalized (25, 32%) and 3 died (4%, all hospitalized). Forty-eight subjects (61%) recovered fully from the infection, whereas 28 (35%) recovered with sequelae. COVID-19 diagnosis was WHO “confirmed” in 65 subjects (82%) and “probable,” supported by a CT scan or chest x-ray, in 6 (8%). Eight patients (10%) had a “suspected” diagnosis.^5^ COVID-19 symptoms were present in 75 patients (95%). As previously reported in PMDs,^8^ the most common symptoms comprised fever (52, 66%), fatigue (51, 65%), persistent cough (39, 49%), headache (33, 42%), myalgia (39%), anosmia/hyposmia (24, 30%), and dysgeusia (23, 29%). Only 3 current or former smokers were identified in the cohort. Consequently, further statistical analysis in this subgroup was not possible.

**Table 1:**
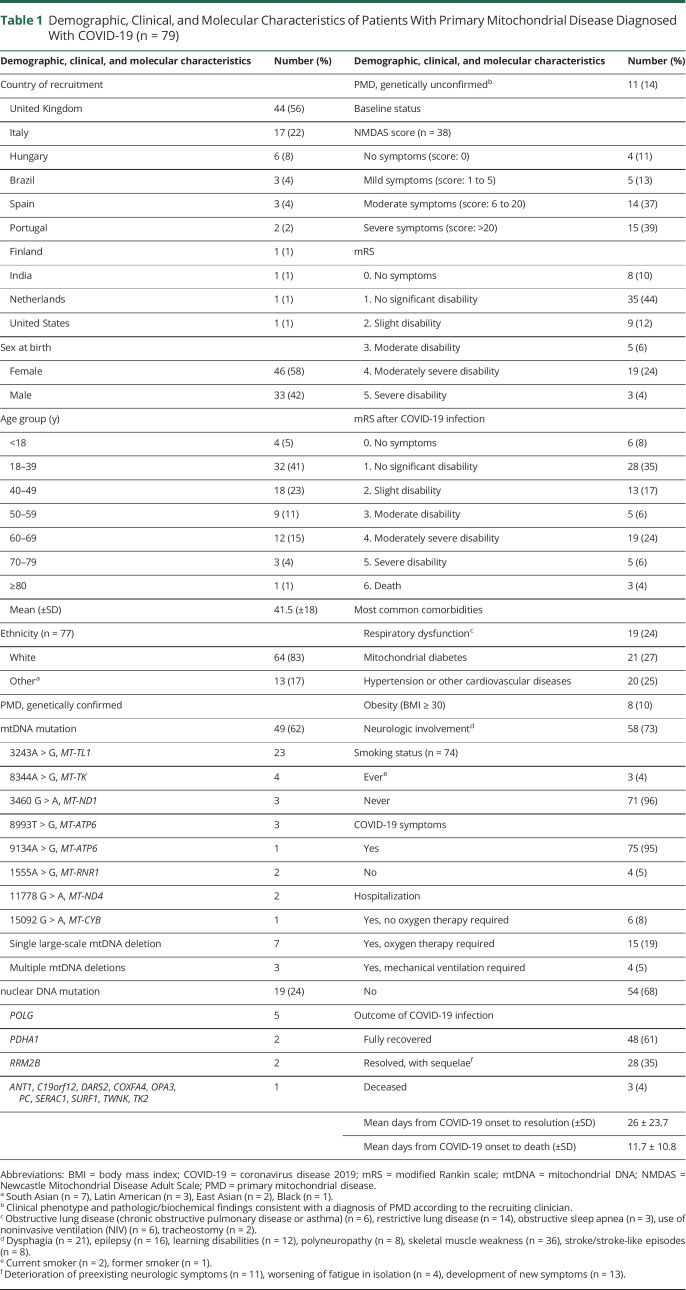
Demographic, Clinical, and Molecular Characteristics of Patients With Primary Mitochondrial Disease Diagnosed With COVID-19 (n = 79)

Neurologic involvement was the most common comorbidity and was present in 58 subjects (73%), followed by mitochondrial diabetes (21%, 27%), hypertension or other cardiovascular diseases (20%, 25%), respiratory dysfunction (19%, 24%), and obesity (8%, 10%). Many patients had multiple single-system and/or multisystem comorbidities. Of those with neurologic problems, 36 had skeletal muscle weakness, 21 dysphagia, 16 epilepsy, 12 learning disabilities, 8 polyneuropathy, and 8 stroke-like episodes (see eTable 1 in the Supplement, links.lww.com/WNL/B828). In patients with respiratory dysfunction, there were 14 subjects with restrictive lung disease, 6 with obstructive lung disease, 6 using noninvasive ventilation, 3 with obstructive sleep apnea, and 2 with a tracheostomy (see eTable 2 in the Supplement).

Univariate analysis (Table 2) showed that hospitalized patients had higher PMD burden (NMDAS score >20, 69%) or moderately severe/severe mRS (52%), compared with people who were not hospitalized (18%, *p* = 0.003, and 17%, *p* = 0.001, respectively). The presence of ≥4 comorbidities was associated with hospitalization (64% vs 28%, *p* = 0.002), in line with previous data from general adult population.^9^ Analysis of individual comorbidities showed that the presence of respiratory dysfunction (56% vs 9%, *p* < 0.001) and neurologic involvement (84% vs 48%, *p* = 0.003) were associated with COVID-19–related hospitalization, and were both present in the 3 deceased subjects. No significant difference in hospitalization status was detected by age, sex, ethnicity, or PMD diagnosis.

**Table 2:**
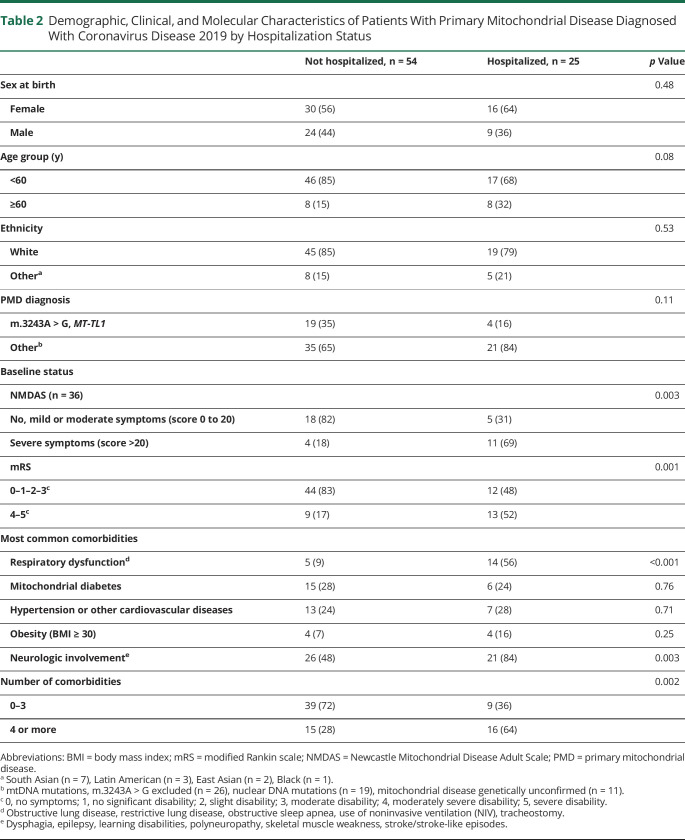
Demographic, Clinical, and Molecular Characteristics of Patients With Primary Mitochondrial Disease Diagnosed With Coronavirus Disease 2019 by Hospitalization Status

Multivariable analysis adjusted for age group, respiratory dysfunction, neurologic involvement, and the mRS, discerned respiratory dysfunction was independently associated with COVID-19 hospitalization (odds ratio [OR], 7.66; 95% CI, 2–28; *p* = 0.002). The results of the sensitivity multivariate are the same when only confirmed cases (n = 65) are included (OR 12.2; CI 2.77–53.77, *p* = 0.001).

## Discussion

To date, this study represents the largest cohort of people with PMDs and COVID-19 ever reported. We have identified a group of patients with PMD who are extremely vulnerable to SARS-CoV-2 because of respiratory dysfunction. Their respiratory dysfunction is an independent risk factor for developing severe COVID-19, and coexisting comorbidities and high disease burden contribute toward the risk of COVID-19–related hospitalization, a factor now recognized in other chronic neurologic disorders.^10^ We could not confirm the incremental impact of age for COVID-19 severity, and so advice for people with PMDs should be adhered to for any age.^11,12^ Ongoing sequelae after resolution of COVID-19 was detected in 35% of our cohort. These included deterioration of preexisting neurologic symptoms (n = 11), worsening of isolated fatigue (n = 4), and development of new symptoms (n = 13). Limitations of this study include the cross-sectional design and voluntary nature of the registry; thus, a selection bias toward severe, rather than asymptomatic and mild, COVID-19 could exist. Furthermore, the limited sample size does not allow outcomes to be correlated with specific genotypes or comorbidities.

In conclusion, the data presented here were collected during the initial stages of the COVID-19 pandemic and were not available to influence clinical decision making during the initial wave. However, we consider that in future waves of COVID-19, or similar pandemics, this information will be crucial to clinicians worldwide managing patients with PMDs and other conditions where there is mitochondrial dysfunction and significant respiratory muscle weakness.
